# The real-world clinical effectiveness of durvalumab in advanced biliary tract cancer: a mimic comparative analysis through survival data reconstruction

**DOI:** 10.3389/fimmu.2025.1643844

**Published:** 2025-11-18

**Authors:** Hong-xiang Ji, Ma-Hui Si, Zhe Sun, Ning Yang, Zhan Chen

**Affiliations:** 1 Department of General Surgery, The Chenggong Hospital (the 73rd Group Military Hospital of PLA), Xiamen University, Xiamen, Fujian, China; 2 Fifth Department of Hepatobiliary Surgery, The Eastern Hepatobiliary Surgery Hospital, Second Military Medical University (Navy Medical University), Shanghai, China

**Keywords:** biliary tract cancer, durvalumab, immunotherapy, gemcitabine-cis platin, real-world

## Abstract

**Background:**

The TOPAZ-1 study results represented significant advancement in the treatment of advanced biliary tract cancer (BTC) by combining durvalumab with gemcitabine–cisplatin (DGC). However, the highly selected patient population may not reflect the real-world scenarios. To gain deeper insights into this combination regimen, we conducted an evidence collection and a mimic survival comparative analysis.

**Methods:**

Records were identified through a formal search of PubMed and Web of Science. Six retrospective cohort studies with real-world evidence were definitively included. The individual patient data for OS and PFS were reconstructed and analyzed. The outcomes different from TOPAZ-1 were summarized and compared.

**Results:**

Whether Asia or non-Asia group, the mOS was similar to the TOPAZ-1 (Asian group: 12.57 months vs. TOPAZ-1, HR = 0.91, 95% CI: 0.69-1.21, log rank P = 0.53; non-Asian group: 13.61 months vs. TOPAZ-1, HR = 1.10, 95% CI: 0.91-1.31, log rank P = 0.323). The mPFS for the Asian group did not show significant differences compared with TOPAZ-1 (5.63 months vs. TOPAZ-1, HR = 1.09, 95% CI: 0.88-1.35, log rank P = 0.422), whereas for the non-Asian group differences exist (6.58 months vs. TOPAZ-1, HR = 0.80, 95% CI: 0.70-0.92, log rank P = 0.002), but potentially influenced by patient ethnicity. The disease control rate in the real world was not so favorable as that in TOPAZ-1. The most common adverse events (AEs) in real-world scenarios were fatigue (26.01%), leukopenia (24.64%), anemia (24.30%), and thrombocytopenia (21.14%). The incidence of immune-related AEs of grades 3–4 was slightly higher in the real world compared with TOPAZ-1 (4.0% vs. 2.4%). Factors such as ECOG-PS, age, alternative doses of durvalumab, neutrophil-to-lymphocyte ratio (NLR), baseline CEA levels, baseline CA19–9 levels, and metastatic disease could be prognostic factors under DGC regimen, with NLR showing a potential as a predictive marker for survival benefit.

**Conclusions:**

The efficacy and safety of the DGC regimen for patients with advanced BTC are confirmed through a comparative analysis and aggregation of real-world evidence in this study. Further real-world investigations are still warranted to determine if the DGC regimen has a broader therapeutic indication and to identify predictive markers for survival benefit. Efforts are required to improve the cost-effectiveness of the DGC regimen to facilitate its wider and standardized use.

## 1 Introduction

Biliary tract cancer (BTC) encompasses a group of highly aggressive malignant tumors originating from the biliary tree, including intrahepatic duct, extrahepatic bile duct, and gallbladder carcinoma (GBC) (1). Extrahepatic CCAs (eCCAs) are further classified into perihilar and distal CCAs according to anatomical location (2). Recent data indicate a rise in the global incidence (3–5), accompanied by a poor prognosis that 5-year overall survival rates range from 5% to 15% (6, 7). There is significant geographic variation in the incidence of BTC and its subtypes. In the Western world, intrahepatic cholangiocarcinoma (iCC) is the most common subtype of BTC, whereas in India, GBC is the most prevalent subtype, accounting for 10% of the global GBC burden (8). The only curative option is surgery followed by adjuvant chemotherapy, but only 20% patients with BTC are eligible for surgery at the time of diagnosis (9, 10). For the treatment for advanced BTC, gemcitabine was first established as a valid option in 1996. Then in 2010, the phase III study ABC-02 trial investigated gemcitabine in combination with cisplatin (GC) against gemcitabine alone, demonstrating a statistically significant improvement in median overall survival (11.7 vs. 8.1 months). This established a new standard of care for advanced BTC. Nevertheless, the predicted 24-month survival rate was only 15% (11).

In the past decade, treatment for advanced BTC remained unchanged. Until 2022, the TOPAZ-1 study results introduced a breakthrough. The combination of durvalumab with chemotherapy (durvalumab plus gemcitabine–cisplatin, DGC) first achieved a median overall survival exceeding 1 year, along with improved progression-free survival (PFS) and higher objective response rates (ORR) to therapy (12). The overall survival (OS) benefit with DGC was seen across all clinically relevant subgroups, and the 24-month overall survival rate with DGC was approximately doubled compared with placebo plus GC in the participants with a complete or partial response and for those with stable disease (13). Then, the European Medicines Agency (EMA) and the United States (US) Food and Drug Administration (FDA) approved DGC as the new first-line standard of care for patients with untreated, metastatic, or unresectable BTC.

However, due to the highly selected patient population enrolled in the TOPAZ-1 trial, it may not reflect the real-world clinical scenarios. During or after the TOPAZ-1 study, multiple centers worldwide actively attempted to implement this treatment regimen. Following the release of the TOPAZ-1 results, various real-world clinical outcomes were reported and compared with those from TOPAZ-1. However, international collaboration in this field remains limited. Therefore, this study gathered results from recent global real-world clinical studies on durvalumab plus chemotherapy, reconstructed the patient survival data by utilizing their KM curves, and conducted simulation comparative analysis. The aim is to provide more real-world insights into the effectiveness of durvalumab in the treatment of advanced BTC.

## 2 Methods

Study selection

In this mimic comparative analysis by survival data reconstruction, a total of 11 studies were initially collected. A systematic search was conducted on PubMed and Web of Science from June 1, 2022 (the publication date of the TOPAZ-1 results), to April 30, 2025. Search terms included “durvalumab”, “real-world or real life”, “biliary tract cancer or BTC or cholangiocarcinoma”, “gallbladder cancer”, “unresectable or metastatic or advanced”. Each record was screened by two authors (Hong-xiang Ji and Ma-Hui Si). Considering the duplicate case information, four previous reports from a same author (Rimini M) were excluded. Another report was excluded due to an unclear treatment regimen, resulting in the final inclusion of 6 reports. This study was conducted following the Preferred Reporting Items for Systematic Reviews and Meta-Analyses (PRISMA) reporting guideline for individual patient data (IPD). (14, **Supplementary 1**).

### 2.1 Quality assessment and IPD extraction from reported Kaplan–Meier curves

The risk of bias among the trials was evaluated by Hong-xiang Ji and Ma-Hui Si using the Cochrane Risk of Bias Tool and Newcastle–Ottawa Scale (NOS) (15). Disagreements were discussed and resolved by a third reviewer (Ning Yang). The OS and PFS Kaplan–Meier curves and the number at risk data from the durvalumab plus chemotherapy arm were extracted from the eligible trials. Since one report (16) has not provided the number at risk data, which was important for survival data reconstruction, we decided to exclude it from “IPD from KM” extraction but still collected its positive outcomes.

### 2.2 Reconstruction of survival data and mimic comparative analysis

The methods for data reconstruction referred to the report from Liu et al. and utilized the Shiny application (https://www.trialdesign.org/one-page-shell.html# IPDfromKM) following its user guide (17). The quality of the reconstructed patient-level data was assessed by inspecting the shape of the survival curves, survival outcomes, survival rates, and hazard ratios (HRs). The IPD data reconstruction process was repeated more than three times by two different authors (Hong-xiang Ji and Ma-Hui Si) for each report to obtain a dataset which can best reproduce the original results. The final version of the dataset was determined by a third author (Zhe Sun) based on quality assessment, and then the necessary data synthesis for the study was performed. Mimic survival comparative analyses were also conducted using the Shiny application. A P value < 0.05 was considered statistically significant.

## 3 Results

**Figure 1:**
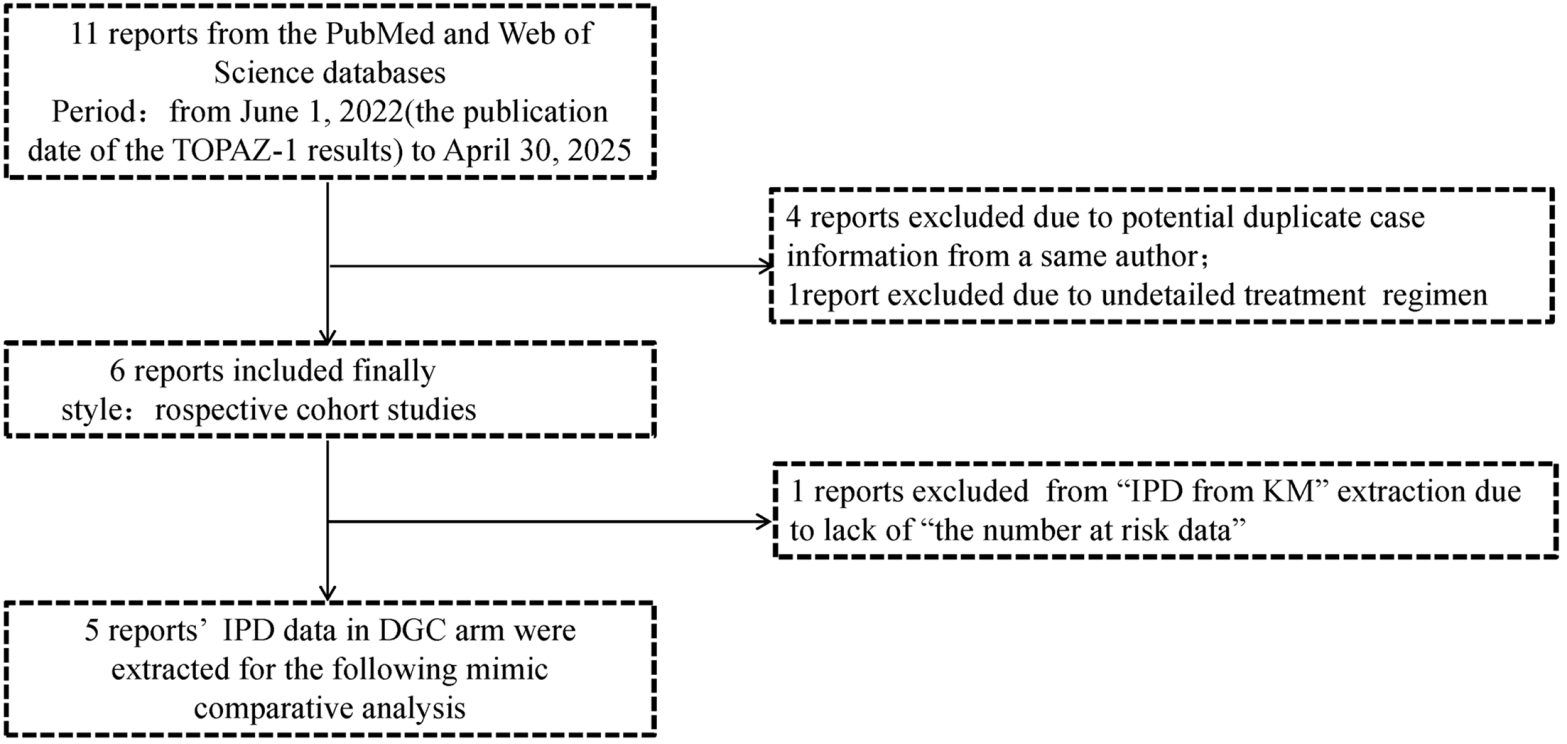
The strategy of reports collection.

Through systematic searching, 11 reports (8, 16, 18–26) were initially collected from the PubMed and Web of Science databases, but we found that five in these reports were authored by a same researcher from Rimini M, Italy. After careful screening, we excluded four (18, 19, 22, 23) of these five reports due to potential duplicate case information, retaining the one (20) with the largest case records and the most recent publication date. One more report (24) was excluded due to undetailed treatment regimen. Ultimately, six reports were included in this study; the basic characteristics of these reports are listed in **Table 1**. All of them were retrospective cohort studies and published in 2024. Within them, five reports rated as high-quality (≥7 stars) (NOS scores ranged from 5 to 9) and all clearly defined exposure (**Supplementary 2**). During the IPD data reconstruction process, we found one additional report (Olkus A et al., 2024) that did not provide the number at risk data, which led us to decide to exclude it from “IPD from KM” extraction but still collected its positive outcomes. The strategy of report collection is shown in **Figure 1**.

**Table 1:** Basic characteristics of the enrolled reports.

Num	Study	Region and center counts	Study type	Period of case data	Median follow-up time(m)	Case(n)	Gender (male/ female)	Median age (y)	Primary tumor position(n)			The real-scenarios of treatment	Reason for the difference in treatment regimen
									ICC	eCCAs	GBC		
B	Mitzlaff et al. (2024) (25)	German-9	Retrospective cohort study	2021- 2024	9 (95% CI, 7.6-10.4)	165^*1^(134)	86/79	63	100	47	18	1.The majority of patients (134/165 = 81.2%) received DGC treatment as first‐line regimen; 2. 116 patients (70.3%) were not treated per TOPAZ‐1 protocol: 12 patients (7.2%) received > 8 cycles and 104 patients (63.0%) received < 8 cycles.	Durvalumab was added during the treatment course after its’ approval by the EMA
C	Muddu et al. (2024) (8)	India-14	Retrospective cohort study	2020.07- 2023.07	6.8 (95% CI, 5.9-7.8)	148	86/62	57.5	41	13	94	1.The majority of patients (134/148 = 90.5%) received durvalumab plus chemotherapy (mostly gemcitabine based) as the first line of treatment. 2.The most common dosing schedule of durvalumab was 1,500 mg (n=132; 89%), but 10.8% of patients received a dose lower than 1,500 mg, most commonly at 500 mg once every 3 weeks.	For logistical and cost Reasons (more than 70% of patients bore out-of-pocket expenses for durvalumab).
E	Reimann et al. (2024) (26)	Austria -5 Germany-1	Retrospective cohort study	2022.04- 2024.01	9.34 (95% CI, 6.58-11.18)	102 (96)	58/44	65.18	62	27	13	1.Carboplain/gemcitabine/durvalumab in 5 cases 2.Oxaliplatin/gemcitabine/durvalumab in 1 case	/
F	Huang et al. (2024) (21)	Taiwan-2	Retrospective cohort study	2021.08- 2023.06	7.9 (95% CI, 4.7 - 12.2)	45	20/25	59.9	31	7	7	1.Oxaliplatin/gemcitabine/durvalumab in 2 cases 2.The median durvalumab dose received was 1440 mg (IQR 1,000-1,440 mg).	The expenses of durvalumab is not fully covered by Taiwan’s National Health Insurance program, the dosage can vary depending on each patient’s financial situation (patients must pay out-of-pocket).
G^*2^	Olkus et al. (2024) (16)	Heidelberg, Germany -single	Retrospective cohort study	2022.04- 2023.09	6.2 (range 1–14.7)	35	15/20	62	21	8	6	Same as TOPAZ-1	/
H	Rimini et al. (2024) (20)	39 sites in 11 countries	Retrospective cohort study	2022.02- 2024.01	8.5 (95% CI, 7.9–9.5)	666	355/311	67	363	168	135	Same as TOPAZ-1	/
sum	/	/	/	/	/	1161	620/541	/	618	270	273	/	/

*1: 134 patients used the DGC regimen as their first-line treatment option; the IPD data were extracted from them. *2: The number at risk data not provided.

Before conducting the mimic survival comparisons, the patient-level data in the durvalumab plus chemotherapy arm from the TOPAZ-1 and the five included reports were reconstructed first to confirm the feasibility of the methods used in this analysis. For TOPAZ-1, the reconstructed median OS was 12.82 months (95% CI, 11.25-14.23) (original: 12.8 months [95% CI, 11.1-14.0]) and median PFS 7.26 months (95% CI, 6.78-7.44) (original: 7.2 months [95% CI, 6.7-7.4]). The comparison of the reconstructed data with the original outcomes for the five included reports were shown in **Table 2**. Based on this results, our reconstructed Kaplan–Meier survival curves demonstrated satisfactory repeatability compared with the original curves and datasets.

**Table 2:** Comparison of the reconstructed outcome data with the original data.

Num	Study	Original-DGC arm		Reconstructed-DGC arm	
		mOS (months)	mPFS (months)	mOS (months)	mPFS (months)
TOPAZ-1		12.8 (95% CI, 11.1-14.0)	7.2 (95% CI, 6.7-7.4)	12.82 (95% CI, 11.25-14.23)	7.26 (95% CI, 6.78-7.44)
B	Mitzlaff et al. (2024) (25)	14 (95% CI, 11.1-16.9)	8 (95% CI, 6.8-9.2)	14.05 (95% CI, 12.04-NA)	8.07 (95% CI, 7.02-10.04)
C	Muddu et al. (2024) (8)	12 (95% CI, 7.8-16.3)	8.2 (95% CI, 7.1-9.4)	12.14 (95% CI, 9.78-NA)	8.33 (95% CI, 6.32-9.69)
E	Reimann et al. (2024) (26)	13.61 (95% CI, 11.28-21.63)	6.51 (95% CI, 4.77-7.27)	13.61 (95% CI, 12.17-NA)	6.58 (95% CI, 5.05-7.39)
F	Huang et al. (2024) (21)	15.8 (95% CI, 7.9-23.8)	5.6 (95% CI, 4.4-6.9)	15.84 (95% CI, 9.58-NA)	5.63 (95% CI, 4.92-9.58)
H	Rimini et al. (2024) (20)	15.1 (95% CI: 13.4-29.1)	8.2 (95% CI: 7.5-8.9)	15.10 (95% CI: 13.54-18.42)	8.24 (95% CI: 7.66-8.94)

### 3.1 Mimic comparative analysis by survival data reconstruction

Among the studies included, the cases reported in B, E, and H primarily comes from non-Asian regions (Germany, Austria, Italy), and those in C and F are mainly from Asian regions (India, Taiwan), and the “number at risk time” intervals differ, being 5 and 6 months, respectively. At the same time, the etiological and genomic differences between Asian and non-Asian BTC patients were considered. For example, hepatitis B and biliary stones are the high-risk factors for Asian patients whereas metabolic syndrome, hepatitis C, and alcoholism are the high-risk factors for European and American patients. The IDH1 mutation in European and American patients with iCC was higher than that in Asian patients (37, 38). Ultimately, this study combined B, E, and H into one group (non-Asian group), and C and F into another (Asian group), for data integration.

**Figure 2:**
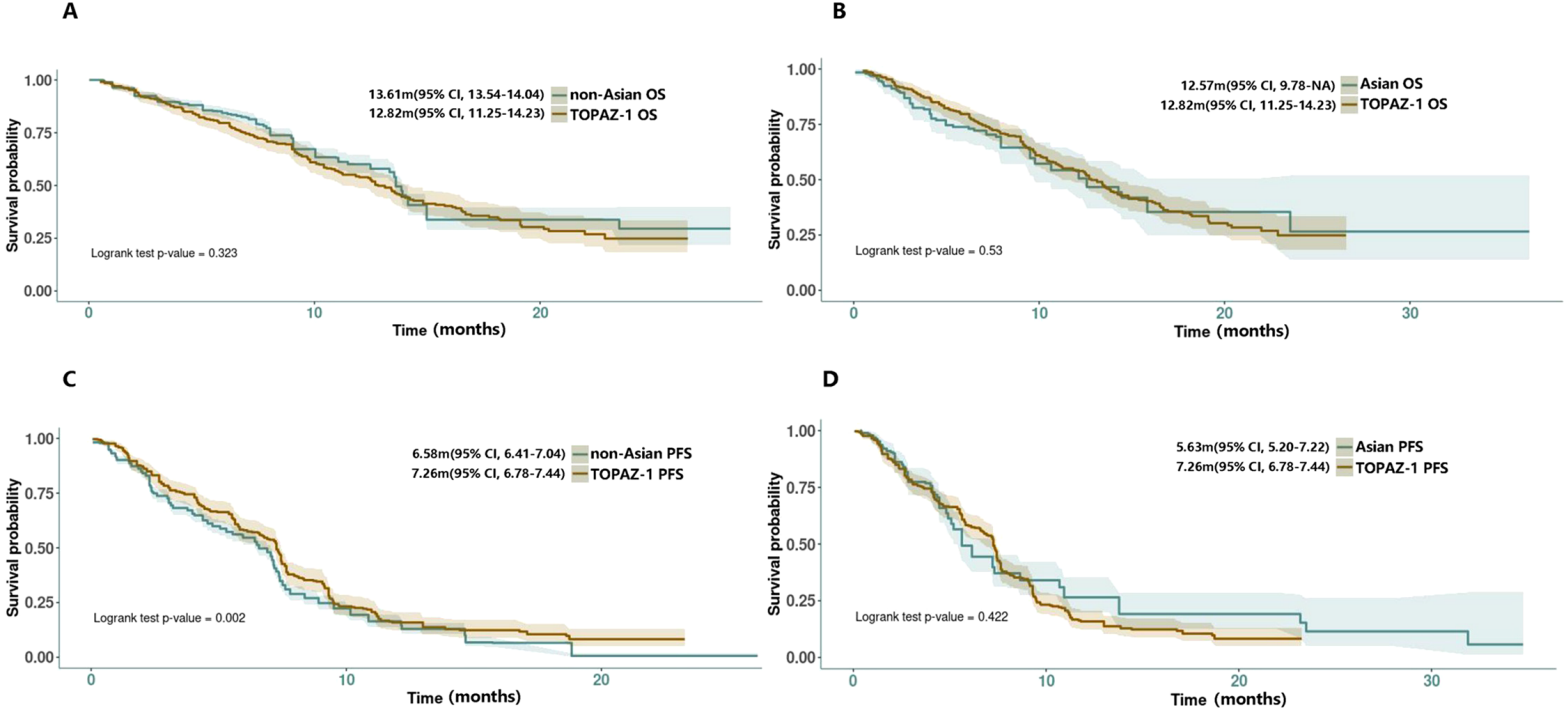
**(A, B)** Reconstructed OS curves for comparison between non-Asian/Asian group with TOPAZ-1 DGC arm. **(C, D)** Reconstructed PFS curves for comparison between non-Asian/Asian groups with the TOPAZ-1 DGC arm.

The two group’s survival data in DGC arm were reconstructed and then compared with the DGC arm from the TOPAZ-1 study. The results showed that the reconstructed median overall survival (mOS) for the non-Asian group was 13.61 months (95% CI: 13.54-14.04), whereas for the Asian group it was 12.57 months (95% CI: 9.78-NA). There was no significant difference between the two groups compared with the TOPAZ-1 study regarding mOS (non-Asian group mOS vs. TOPAZ-1 mOS: HR = 1.10, 95% CI: 0.91-1.31, log rank P = 0.323; Asian group mOS vs. TOPAZ-1 mOS: HR = 0.91, 95% CI: 0.69-1.21, log rank P = 0.53) (**Figures 2A, B**). The reconstructed median progression-free survival (mPFS) for the non-Asian group was 6.58 months (95% CI: 6.41-7.04), which was statistically different from the DGC arm in the TOPAZ-1 study (non-Asian group mPFS vs. TOPAZ-1 mPFS: HR = 0.80, 95% CI:0.70-0.92, log rank P = 0.002). For the Asian group, the mPFS was 5.63 months (95% CI: 5.20-7.22), but there was no statistical difference compared with the DGC arm in the TOPAZ-1 study (Asian group mPFS vs. TOPAZ-1 mPFS: HR = 1.09, 95% CI: 0.88-1.35, log rank P = 0.422) (**Figures 2C, D**).

In the TOPAZ-1 study, the observed benefits in overall survival and progression-free survival with DGC remained generally consistent across relevant subgroups based on primary tumor location (iCCAs, eCCAs, GBC). However, some real-world findings have shown different results. In Mitzlaff et al. (2024) (25), they found that survival was significantly shorter for GBC patients with a median OS of 9 months (95% CI, 5.5-12.4; p=0.02) in comparison with patients with iCC and/or eCCAs (mOS not reached). The mPFS for patients with GBC was 6 months (95% CI, 4.6-7.4), also shorter than that for patients with iCC at 7 months (95% CI, 5.8-8.1) and/or eCCAs at 13 months (95% CI, 10.5-15.5; p=0.015). In Muddu et al. (2024) (8), they also observed a trend of lower mOS in the GBC subgroup compared with the remaining subgroups (mOS: iCC 14.2months [95% CI, 9.8-18.6] vs. eCCAs [not reached] vs. GBC 12.1 [95% CI, 8.8-15.3]), although not statistically significant (p=0.381).

### 3.2 The ORR, SDR, and DCR of the DGC treatment

**Table 3** illustrates the ORR (overall response rate), SDR (stable disease rate), and DCR (disease control rate) of the DGC arm in real-world reports. It is evident that in real-world clinical scenarios, the DCR was not so favorable as it is in the TOPAZ-1 trial. However, in the TOPAZ-1 trial, the overall survival, including long-term survival, improved for participants who achieved disease control in the DGC arm, whether CR (complete response) or PR (partial response) or SD (stable disease). The results from real-world reports are largely consistent with this and even showed more pronounced outcomes. In Muddu et al. (2024) (8), the mOS in patients who achieved clinical benefit was 23.1 months (95% CI, 10.2-36), obviously longer than 7.6 months (95% CI, 1.6-13.5) in patients who had PD (progressive disease) during treatment (p=0.002). In Reimann et al. (2024) (26), the patients who achieved at least a PR got a mOS of 14.14 months (95% CI, 13.55-NR). For the patients with SD, the mOS was 12.20 months (95% CI, 10.52-NR), and for those with PD, it was 8.52 months (95% CI, 5.46-NR). Patients with CR or PR, demonstrated significantly better overall survival compared with non-responders (HR: 0.29; 95% CI: 0.12-0.72; p=0.007). In Huang et al. (2024) (21), the responders, who had at least a PR or SD≧6 months, had a mOS of 15.8 months, whereas the non-responders, who had SD <6 months or PD, had a mOS of only 3.3 months.

**Table 3:** ORR, SDR^*1^, and DCR of the DGC regimen in the real world.

Num	Study	ORR(CR+PR)%	SDR%	DCR%
TOPAZ-1^*2^		26.7	58.6	85.3
B	Mitzlaff et al. (2024) (25)^*3^	31	36	67
C	Muddu et al. (2024) (8)	29.7	24.4	54.1
E	Reimann et al. (2024) (26)	35.11	34.46	71.57
F	Huang et al. (2024) (21)	31.1	40	71.1
G	Olkus et al. (2024) (16)	14.7	47	61.7
H	Rimini et al. (2024) (20)	32.6	45.2	77.8

*1:SDR:stable disease rate.

*2:The information from the initial report (12).

*3:In the 134 cases treated with DGC as first-line therapy.

Additionally, in Mitzlaff et al. (2024) (25), the ORR and DCR of patients who received DGC regimen as the first‐line treatment (n=134, ORR = 31%, DCR = 67%) was significantly better in comparison with patients who received it as a second- or later‐line treatment, supporting its upfront use. In a previous report from Rimini, they highlighted a correlation between baseline ALT levels (within normal ranges) and NLR (NLR <3) with better ORR (NLR<3 50.0% vs. NLR≧3 31.0%; normal ALT 40.7% vs. elevated ALT 20.4%) (18). Then, in Rimini et al.’s (2024) (20) latest research, the ECOG-PS, disease status, and absence of drainage or stent were reported also associated with higher ORR to treatment (ECOG-PS 0 39.2% vs. ECOG-PS >0 25.6%; locally advanced disease 39.2% vs. metastatic disease 31.8%; absence of drainage or stent 35.2% vs. with drainage or stent 29.9%), but the primary tumor site had no relationship with ORR.

### 3.3 The AEs in DGC treatment

In TOPAZ-1, compared with placebo plus chemotherapy, the DGC treatment was associated with a similar rate of discontinuations due to adverse events. The observed toxicities with DGC were similar to those with chemotherapy or immunotherapy alone. The most common adverse events seen in the DGC arm were anemia (48.2%), nausea (40.2%), constipation (32.0%), and neutropenia (31.7%). The updated report showed that the rate of immune therapy-related adverse effects (irAEs) in long-term survivors was higher in the DGC arm, but grade 3 or 4 irAEs only occurred in one (1%) of 88 participants.

**Figure 3:**
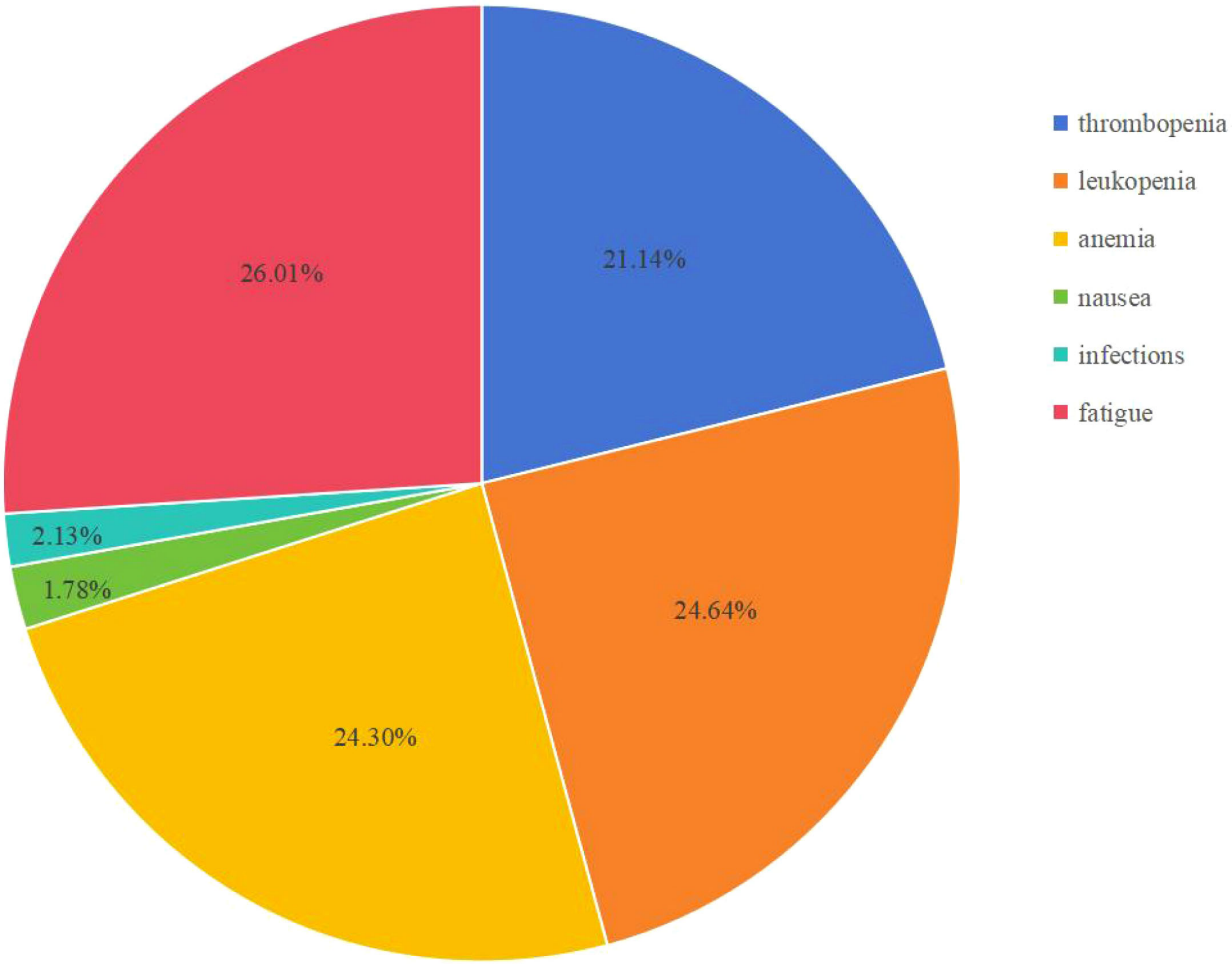
Common AEs in the DGC arm from real-world data.

However, in real-world clinical scenarios, some differences may exist, especially in irAEs (**Table 4**, **Figure 3**). The most common adverse events seen in the DGC arm were fatigue (26.01%), leukopenia (24.64%), anemia (24.30%), and thrombocytopenia (21.14%) calculated based on the reported real-world data. The incidence rate of irAEs (3–4 Grade) calculated based on the reported data in our study is 4.0% (40/998) in the DGC arm. Among included reports, the most common irAEs were skin or thyroid related toxicity, and all 3–4 grade irAEs can be well managed with corticosteroids or immunosuppressants. Considering that the incidence of irAEs was more likely associated with the addition of durvalumab, researchers have also paid attention to the correlation between irAEs and survival outcomes. In Mitzlaff et al. (2024) (25), the occurrence of irAEs was found to be associated with a slight trend of better outcome with a mOS not reached versus 12 months (95% CI, 9.3-14.7; p=0.35) and mPFS of 11 months (95% CI, 3.4-18.5) versus 8 months (95% CI, 7.1-8.9; p=0.401). However, the response rates was not significantly different between patients with irAEs or not.

**Table 4:** The incidence rate of AEs in the real world.

Num	Study	Incidence rate of AE	Incidence rate of AE (3-4 grade)	Incidence rate of irAE	Incidence rate of irAE (3-4 grade)	Most common AEs	Most common irAEs
TOPAZ-1		99.4	75.7	12.7	2.4	Anemia (48.2%) nausea (40.2%) constipation (32.0%) neutropenia (31.7%)	NR^*1^
B	Mitzlaff K et al. (2024) (25)	78.8	35.2	10.3	5.5 (9/165)	Thrombopenia (27.9%) Neutropenia (26.1%) Anemia (25.5%) nausea (18.8%) Infections (15.8%) Fatigue (13.3%)	ir‐hepatitis (3.0%) ir‐dermatitis (2.4%)
C	Muddu et al. (2024) (8)	NR	NR	NR	7.4 (11/148)	NR	Skin toxicity (skin rash grade 1 or 2) (3.4%) Hypothyroidism (4.3%)
E	Reimann et al. (2024) (26)	NR	NR	NR	NR	NR	NR
F	Huang et al. (2024) (21)	NR	NR	NR	NR	NR	NR
G	Olkus et al. (2024) (16)	NR	60.0	NR	11.0 (4/35)	Anemia (23%) Thrombocytopenia (20%) Leukopenia (17%)	NR
H	Rimini^*2^ et al. (2024) (20)	92.9	46.6	20.0	2.5 (16/650)	Fatigue (55.0%), Neutropenia (47.7%), Anemia (46.8%), Thrombocytopenia (39.2%)	Rash (8.2% all grade; 0.3% grade >2) Itching (10.3% all grade; 0.2% grade >2) Hypothyroidism (5.1% all grade; 0.3% grade >2) hyperthyroidism (1.8% all grade; 0% grade >2) Colitis (1.4% all grade; 0% grade >2) ir-pneumonia(0.01% all grade; 0% grade >2) Hypophysitis (0.01% all grade; 0% grade >2)

*1:NR=not reported.

*2:Safety data were available for 650 patients (one center did not gather any safety information).

### 3.4 The prognostic factors for OS and PFS

In TOPAZ-1, the PD-L1 status (patients with a PD-L1 tumor area positivity [TAP] score of 1% or greater) has not emerged as a significant indicator for clinical prognosis. In the updated result, it was found that among long-term survivors in the DGC arm, more patients (≧10%) had recurrent disease at baseline, an NLR (neutrophil to lymphocyte ratio)≦3, a CA-199 (cancer antigen 199)≦500U/mL, or a CEA (carcinoembryonic antigen)≦5 ng/mL.

**Figure 4:**
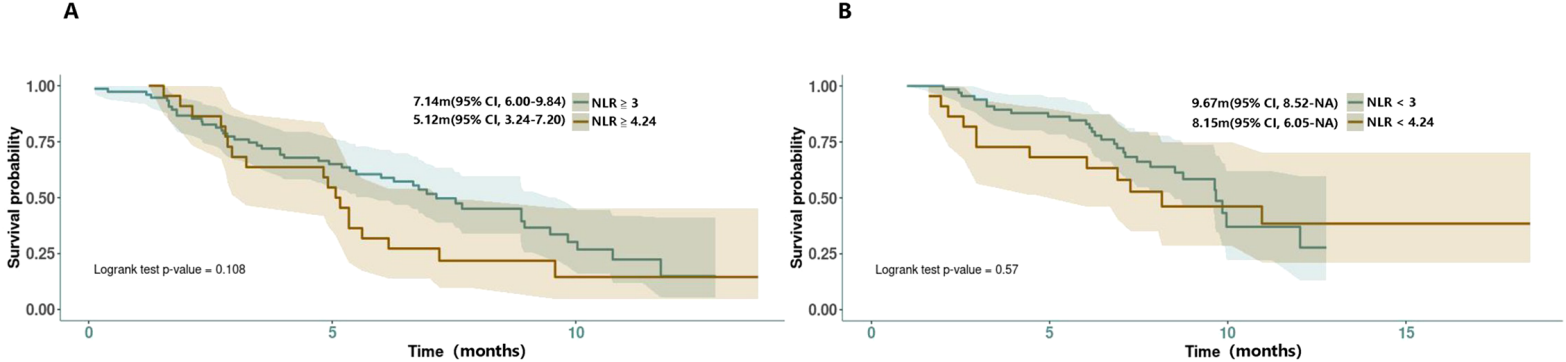
**(A, B)** Reconstructed PFS curves for comparison of the NLR threshold.

In the real-world clinical scenarios, various factors have been reported as valuable references for prognosis (**Table 5**). According to Muddu et al. (2024) (8), factors such as sex, ECOG-PS, BTC subtype, tumor differentiation, durvalumab cycle frequency, chemotherapy dose interruptions, and immunotherapy dose interruptions all did not affect the overall survival, although a lower mOS was observed in the GBC subset. Its multivariable analysis showed that age <60 years and standard dose of durvalumab were associated with improved OS. Patients <60 years had a mOS of 23.5 months compared with 9.5 months for those age ≧ 60 years (P = 0.002). In the study, 10.8% of patients received durvalumab at a dose lower than 1,500 mg (most at 500 mg/3 weeks) due to logistical and cost reasons. The mOS for patients on the standard dose was 12.1 months, compared with 7.5 months for those on alternative doses. In Reimann et al. (2024) (26), age <65 years was significantly associated with improved OS. In Huang et al. (2024) (21), a restricted cubic spline (RCS) analysis suggested a similar efficacy between doses of 1,000 and 1500 mg but potentially worsened at doses less than 1,000 mg, although not statistically significant. They also found a high NLR (≧4.24) to be associated with poor survival outcomes, a finding supported by Rimini et al.’s previous research (18), in which 25.4% of the patients with NLR≧3 had PD compared with 4.5% of patients with NLR<3 (p=0.002), and 23.2% of patients with ECOG PS > 0 had progression versus 8.2% of patients with ECOG PS = 0 (p=0.02) at the first CT scan. As the NLR-related PFS KM curves were provided by Rimini et al. (18) and Huang et al. (21) and with the attempt to find a more credible threshold, we also conducted a reconstruction of NLR-related survival data for comparison (NLR≧4.24 vs. ≧3; NLR≦4.24 vs. ≦3). However, the result showed no statistical significance between them (NLR≧4.24 vs. ≧3: 5.12m vs. 7.14m, HR = 1.56, 95% CI:0.90-2.70, log rank P = 0.108; NLR≦4.24 vs. ≦3: 8.15m vs. 9.67m, HR = 1.22, 95% CI: 0.61-2.44, log rank P = 0.57) (**Figure 4**). In the latest research from Rimini et al. (2024) (20), factors such as high baseline CA-199, high baseline CEA, and metastatic disease at initial were also found to affect the survival outcomes with statistical significance.

**Table 5:** The prognostic factors for OS and PFS.

Num	Study	Risk factors for OS^1*^	Risk factors for PFS^2*^
B	Mitzlaff et al. (2024) (25)	1.ECOG-PS ≧1 (ECOG 0 vs. ≧1; p=0.022) 2.GBC (GBC vs. other BTCs; p=0.029)	NR
C	Muddu et al. (2024) (8)	1.Age ≧ 60 (age < 60 vs. ≧ 60; p=0.001) 2.Alternative doses of durvalumab (standard dose [1,500 mg] vs. alternative doses; p<0.001)	NR
E	Reimann et al. (2024) (26)	Age ≧ 65 (age< 65 vs. ≧ 65; p=0.009)	NR
F	Huang et al. (2024) (21)	1.ECOG-PS ≧2 (ECOG 0–1 vs≧ 2; p=0.003) 2.NLR≧4.24 (NLR< 4.24 vs≧ 4.24; p=0.004)	1.ECOG-PS ≧2 (ECOG 0-1 vs. ≧ 2; p=0.038) 2.NLR ≧4.24 (NLR< 4.24 vs≧ 4.24; p=0.030) 3.CA-199 ≧92.9U/ml (CA199 <92.9 vs. ≧92.9; p=0.005)
G	Olkus et al. (2024) (16)	NR	NR
H	Rimini^*2^ et al. (2024) (20)	1.High baseline CEA levels (higher than normal vs. normal; p=0.0004) 2.ECOG-PS >0 (ECOG 0 vs. ≧1; p=0.0001) 3.Metastatic disease(vs. locally advanced; p<0.0001) 4.NLR >3 (NLR≦3 vs. >3; p=0.0002)	1.High baseline CA-199 (higher than normal vs. normal; p=0.03) 2.High baseline CEA (higher than normal vs. normal; p=0.02) 3.ECOG-PS >0 (ECOG 0 vs≧1; p<0.0001) 4.Metastatic disease (vs. locally advanced; p=0.0001)

*1&2 After the multivariate analyses for OS/PFS by using Cox regression models.

## 4 Discussion

In TOPAZ-1, the chemoimmunotherapy-DGC significantly prolonged overall survival and progression-free survival with the addition of durvalumab, a human IgG1 monoclonal antibody, and demonstrated a 24% decrease in risk of death at 2 years. A growing body of evidence indicated that cytotoxic chemotherapy has immunomodulatory effects, providing a rationale for chemoimmunotherapy (27, 28). The KEYNOTE-966 study also showed similar results, with the combination of pembrolizumab and chemotherapy (PGC) yielding a mOS of 12.7 months and mPFS of 6.5 months. There is one difference between the two trials; in KEYNOTE‐966, cisplatin was stopped after eight cycles of combined therapy and the duration of gemcitabine was not limited, whereas in the TOPAZ‐1 trial, both cisplatin and gemcitabine were stopped after eight cycles. The DCR in KEYNOTE-966 was lower compared with the TOPAZ-1 trial (54.1% vs. 85.3%), but the ORR was comparable (29.7% vs. 26.7%) (29). Recently, the results from the IMbrave151 trial showed that the effect of atezolizumab, another PD-L1 drug, combined with GC in the treatment of BTC was largely comparable with that of DGC or PGC with respect to mPFS (7.9 months), OS(14.6 months), and ORR (26.5%) (36).

However, the highly selected patient population in TOPAZ-1 may have reduced the representativeness of the real-world scenarios, whereas the international collaboration in this field is still limited. Based on our mimic comparative analysis utilizing available limited real-world data, the efficacy of the DGC regimen in treating advanced BTC is commendable. The survival outcomes from our analysis are similar to those of the TOPAZ-1 study, except for the median mPFS in the non-Asian group (6.58 months), which showed a statistically significant difference compared with the DGC group in the TOPAZ-1 study. This discrepancy may be attributed to the higher proportion of Caucasian patients, which was emphasized in Reimann et al. (2024) (26). Compared with TOPAZ-1, the inclusion criteria for real-world clinical trials were more complex and broad due to some objective reasons, but the reported outcomes still supported the therapeutic value of DGC regimen in advanced BTC. In the early report from Rimini et al. (23), no significant differences were observed between early and late relapse groups (disease recurrence occurring ≦6 months or >6 months after surgery) in OS or PFS, under DGC therapy. In Olkus et al. (2024) (16), 51% patients met the inclusion criteria of the TOPAZ-1 trial (TOPAZ-1 IN group), whereas the remaining 49% did not (TOPAZ-1 OUT group). The most common reason for the TOPAZ-1 OUT group was preexisting autoimmune-associated disorders, followed by ECOG PS > 1. Then, the comparison between the two groups showed no significant difference in OS and PFS (mOS:10.3 vs. 9.7 months; mPFS:5.3 vs. 5 months); the DCR was also similar in both groups (61.1% vs. 58.8%), although the ORR in the TOPAZ-1 IN subgroup was higher (22.2% vs. 5.8%). The inclusion of patients beyond the TOPAZ-1 criteria even did not lead to an increase in AEs or alteration of the DGC treatment. Both Mitzlaff et al. and Rimini et al. found that patients with chronic liver diseases (including chronic viral hepatitis B or C or liver cirrhosis) could get similar outcomes and safety profiles. In the earlier research from Rimini et al., they even noted that the absence of viral infection correlated with a worse PFS (18, 25).

In TOPAZ-1, no clear indication of prognostic risk factors were identified. However, in real-world clinical settings, this issue cannot be overlooked. Among those real-world reports, ECOG-PS, age, alternative doses of durvalumab, NLR, baseline CEA level, baseline CA-199 level, and metastatic disease were found to be factors affecting patients’ OS or PFS. It is noteworthy that NLR has been mentioned in multiple reports, indicating its potential as a predictive marker for patients’ survival benefit under DGC regimen. Previous studies have shown that NLR plays a prognostic role in various solid tumors treated with systemic therapy, particularly ICIs (20). Now, NLR has been widely recognized as a possible surrogate of systemic inflammatory status. Tanaka et al. demonstrated a negative correlation between NLR and CD8+T cells which are crucial for the antitumor immune response (30). More investigations are needed for the confirmation of its predictive role.

In TOPAZ-1, all subgroups could benefitted from the DGC treatment regardless of the primary tumor location. There is also no clear evidence supporting that a specific gene, type of genetic mutation, or TMB (tumor mutation burden) or MSI-H/dMMR status can serve as a therapeutic benefit predictive indicator under a DGC regimen. Unfortunately, in the included real-world studies in our research, no prognostic-related gene markers were identified either. In Mitzlaff et al. (2024) (25), the detected molecular profiles of the cohort matched the known distribution of molecular alterations including FGFR2 alterations (14.6%) and IDH1 or two mutations (14.6%) in iCC, KRAS (44.4%), and TP53 (36.1%) in eCCAs and Her2 (13.3%) in GBC. However, no specific gene mutation among them was found to be a prognostic marker for OS, as well as the microsatellite instability (MSI). In Reimann Pet al. (2024) (26), comprehensive molecular profiling was conducted in 90 patients to explore whether mutations in specific genes or pathways (including insertions, deletions, frameshift alterations, splice site, nonsense, and missense mutations) influenced the response and efficacy of the combined therapy. However, no individual gene or mutation pathway demonstrated a significant association with improved outcome. In Olkus et al. (2024) (16), loss of TP53 (28%), deletion mutations in BRCA1-associated protein 1 (BAP1; 24%), activating mutations of KRAS (21%), deletion mutation of ARID1A (17%), and the TMB of patients were not associated with the response to treatment. A study by Rimini et al. clustered patients based on molecular and genomic alterations, finding that those with alterations in the RTK/RAS pathway and cell-cycle apoptosis had worse outcomes compared with patients with chromatin modification pathway alterations (31), but the sample size was low (n = 51) and further validation on larger and external cohorts are needed.

In our study, DGC treatment-related AEs appeared slightly higher than that in TOPAZ-1 but most seems related to chemotherapy, indicating that durvalumab is indeed a relatively safe medication. Regarding the implementation of the DGC regimen, Olkus et al. found that the frequency of durvalumab cycles did not affect the survival (8), whereas Mitzlaff et al. found that patients who completed eight cycles of DGC regimen (n = 49) showed a similar outcome compared with patients who received more than eight cycles (n = 12) but was significantly better than those received less than eight cycles (n = 71) (25). In the USA and EU, the recommended dosage of durvalumab is 1,500 mg without reduction or escalation. In patients who weigh <30 kg (USA) or ≦36 kg (EU), the recommended dosage is 20 mg/kg. The terminal half-life of durvalumab, based on clearance at baseline, is 18–21 days. There were no clinically significant differences in durvalumab pharmacokinetics based on age (18–96 years), body weight (31–175 kg), gender, or race. Serum levels of albumin (4-57g/L), lactate dehydrogenase (18-15,800 U/L), soluble PD-L1 (67-3,470 pg/mL), tumor type (non-small cell lung, small cell lung, biliary tract, and hepatocellular cancers), or ECOG-PS also did not affect the pharmacokinetics of durvalumab (32) (one possible reason for the inability to identify prognostic markers for durvalumab). On the other hand, in Huang et al. (2024) (21), the pharmacodynamic evidence showed that a 10-mg/kg Q2W dose of durvalumab leads to approximately 93.3% of patients achieving complete suppression of sPD-L1. Moreover, the pharmacokinetic analyses revealed a linear dose–response relationship at doses over 3 mg/kg Q2W, which can ensure the consistent drug exposure. Transitioning to a flat dosing regimen, this corresponds to approximately 15 mg/kg Q3W, or around 900–975 mg. These findings highlight the importance of maintaining a dosage of at least 1,000mg Q3W for clinical efficacy.

For advanced BTC, the cost-effectiveness of durvalumab may hinder its wider and standardized use in real-world scenarios. Zhao et al. conducted a cost-effectiveness analysis for the DGC regimen and found that it was not cost-effective for advanced BTC regardless of receiving or not receiving charitable assistance, unless the price of durvalumab fell by more than 94.2% to less than $0.33/mg (33). Kashiwa et al. found in their analysis that both DGC and PGC (pembrolizumab with gemcitabine–cisplatin) were not cost-effective for Japanese patients compared with the combined chemotherapy regimen-GCS (gemcitabine, cisplatin plus S-1) (35). Ye et al. also found that even for the US patients, the DGC regimen did not offer a cost-effective advantage at current prices (for US:11,730 dollar per cycle), only when the price of durvalumab fell by 67.4% or more (34).

Of course, in addition to chemical immunotherapy, many therapeutic strategies are under active research, for example, the optimized systemic chemotherapy, targeted drugs, ICI-targeted drug combination strategy, double-ICI combination strategy, CAR-T, and tumor vaccine. It is believed that BTC patients will have more effective treatment options in the future.

## 5 Limitations

Several limitations exist in the analysis. Firstly, due to the recent time of release of TOPAZ-1 results, the number of included studies is limited. Then, although there was a low risk of bias among the enrolled trials (**Supplementary 2, 3**), due to the nature of real-world evidence (RWE), all included studies were all retrospective cohort studies, and inherent heterogeneity may be inevitable, for example, differences in treatment implementation due to real-world objective factors and the expansion of patient inclusion criteria. Due to the incomplete availability of KM curves and no at-risk data provided by included studies, there is a pity that further reconstruction and comparison cannot be conducted between subgroups based on points of interest, such as primary tumor location or region/race. Because of the inability to reconstruct response and AE data, only comparison by outcome summarize was conducted in the relevant sections.

## 6 Conclusions

Based on the mimic comparative analysis results and the aggregation of real-world evidence in this study, the efficacy and safety of the DGC regimen for patients with advanced BTC are reaffirmed. Further real-world investigations are still warranted to determine if the DGC regimen has a broader therapeutic indication. Currently, there are no clear indicators for predicting the treatment benefits of DGC regimen. NLR has the potential to become a useful and convenient indicator. In real-world clinical scenarios, the improved cost-effectiveness of the DGC regimen will facilitate its wider application.

## Data Availability

The original contributions presented in the study are included in the article/**Supplementary Material**. Further inquiries can be directed to the corresponding authors.
