# Supplementary material for: The real-world clinical effectiveness of durvalumab in advanced biliary tract cancer: a mimic comparative analysis through survival data reconstruction
Source: Front Immunol. 2025 Nov 18;16:1643844. doi: 10.3389/fimmu.2025.1643844 (PMC12669214; doi:10.3389/fimmu.2025.1643844)
Supplement: Supplementary file 1 [file DataSheet1.pdf]

	Random sequence generation (selection bias)	Allocation concealment (selection bias)	Blinding of participants and personnel (performance bias)	Blinding of outcome assessment (detection bias)	Incomplete outcome data (attrition bias)	Selective reporting (reporting bias)	Other bias
Huang WK 2024	?	+	?	?	+	+	+
Mitzlaff K 2024	+	+	+	?	+	+	+
Muddu VK 2024	?	+	−	?	+	+	+
Olkus A 2024	+	+	?	?	+	+	+
Reimann P 2024	?	−	+	?	+	+	+
Rimini M 2024	+	+	?	−	+	+	+

Newcastle-Ottawa Scale, NOS

Athuor	Representativeness of the exposed cohort	Selection of the non-exposed cohort	Ascertainment of exposure	Demonstration that outcome of interest was not present at start of study	Comparability of cohorts on the basis of design or analysis	Assessment of outcome	Was follow-up long enough for out comes	Adequacy of follow up of cohorts	Total scores
Mitzlaff K, 2024 (25)	★	★	★	★	★	★	★	★	8
Muddu VK, 2024 (8)	★	★	★	★	★	★	☆	★	7
Reimann P, 2024 (26)	★	★	★	★	★	★	★	★	8
Huang WK, 2024 (21)	★	★	★	★	★	★	★	★	8
Olkus A, 2024 (16)	★	★	★	★	☆	★	☆	★	6
Rimini M, 2024 (20)	★	★	★	★	★★	★	★	★	9
